# DR15-DQ6 remains dominantly protective against type 1 diabetes throughout the first five decades of life

**DOI:** 10.1007/s00125-021-05513-4

**Published:** 2021-07-16

**Authors:** Nicholas J. Thomas, John M. Dennis, Seth A. Sharp, Akaal Kaur, Shivani Misra, Helen C. Walkey, Desmond G. Johnston, Nick S. Oliver, William A. Hagopian, Michael N. Weedon, Kashyap A. Patel, Richard A. Oram

**Affiliations:** 1 Institute of Biomedical and Clinical Science, University of Exeter Medical School, Exeter, UK; 2 Department of Diabetes and Endocrinology, Royal Devon and Exeter NHS Foundation Trust, Exeter, UK; 3 Faculty of Medicine, Imperial College, London, UK; 4 Pacific Northwest Research Institute, Seattle, WA USA; 5 Renal Department, Royal Devon and Exeter NHS Foundation Trust, Exeter, UK

**Keywords:** Adult onset type 1 diabetes, DR15-DQ6, HLA, Genetic predisposition, Genetic protection, Genetic resistance, Type 1 diabetes

## Abstract

**Aims/hypothesis:**

Among white European children developing type 1 diabetes, the otherwise common HLA haplotype DR15-DQ6 is rare, and highly protective. Adult-onset type 1 diabetes is now known to represent more overall cases than childhood onset, but it is not known whether DR15-DQ6 is protective in older-adult-onset type 1 diabetes. We sought to quantify DR15-DQ6 protection against type 1 diabetes as age of onset increased.

**Methods:**

In two independent cohorts we assessed the proportion of type 1 diabetes cases presenting through the first 50 years of life with DR15-DQ6, compared with population controls. In the After Diabetes Diagnosis Research Support System-2 (ADDRESS-2) cohort (*n* = 1458) clinician-diagnosed type 1 diabetes was confirmed by positivity for one or more islet-specific autoantibodies. In UK Biobank (*n* = 2502), we estimated type 1 diabetes incidence rates relative to baseline HLA risk for each HLA group using Poisson regression. Analyses were restricted to white Europeans and were performed in three groups according to age at type 1 diabetes onset: 0–18 years, 19–30 years and 31–50 years.

**Results:**

DR15-DQ6 was protective against type 1 diabetes through to age 50 years (OR < 1 for each age group, all *p* < 0.001). The following ORs for type 1 diabetes, relative to a neutral HLA genotype, were observed in ADDRESS-2: age 5–18 years OR 0.16 (95% CI 0.08, 0.31); age 19–30 years OR 0.10 (0.04, 0.23); and age 31–50 years OR 0.37 (0.21, 0.68). DR15-DQ6 also remained highly protective at all ages in UK Biobank. Without DR15-DQ6, the presence of major type 1 diabetes high-risk haplotype (either DR3-DQ2 or DR4-DQ8) was associated with increased risk of type 1 diabetes.

**Conclusions/interpretation:**

HLA DR15-DQ6 confers dominant protection from type 1 diabetes across the first five decades of life.

**Graphical abstract:**

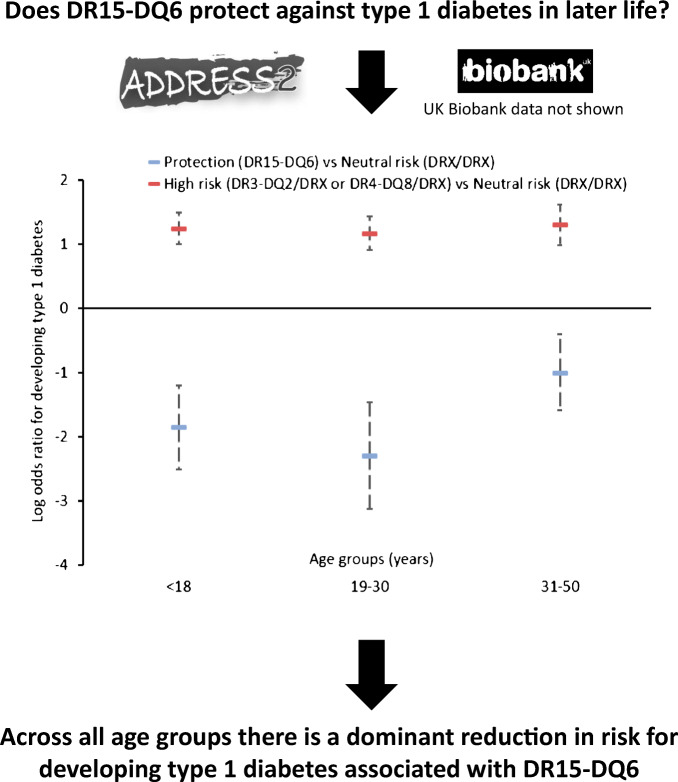

**Supplementary Information:**

The online version contains peer-reviewed but unedited supplementary material available at 10.1007/s00125-021-05513-4.

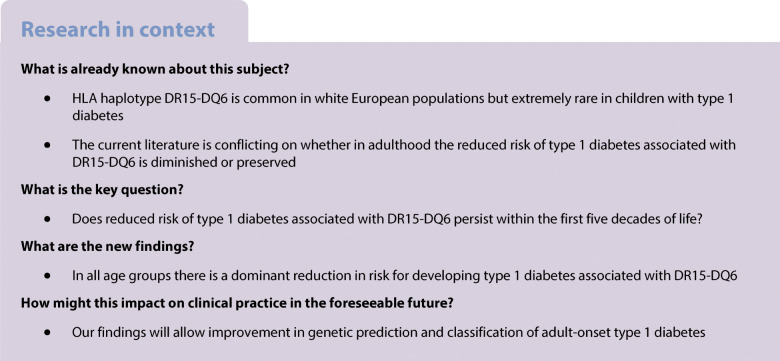



## Introduction

Type 1 diabetes is an autoimmune condition with a strong genetic predisposition. Most of our understanding of the genetic architecture of type 1 diabetes is derived from case–control studies of children and adolescents [1]. These studies have shown most genetic predisposition for type 1 diabetes is associated with a small number of HLA risk alleles including DR3-DQ2 (DRB1*03:01-DQA1*05:01-DQB1*02:01) and DR4-DQ8 (DRB1*04-DQA1*03-DQB1*03:02) [2]. Some HLA alleles are associated with protection from type 1 diabetes. DR15-DQ6 (DRB1*15-DQA1*01-DQB1*06) is a particularly good example as it is common in European populations (frequency ~15–20%) [3, 4] but rare in children and young adults with type 1 diabetes (frequency 1–2%) [2, 5–7].

The interaction between age of onset and genetic protection against type 1 diabetes is not well understood. Population and at-risk relative studies suggest that the protection provided by HLA against type 1 diabetes diminishes with increasing age at diagnosis [8], yet at least one study has shown the opposite [9]. Very few studies of DR15-DQ6 protection in type 1 diabetes have focused on adults in sufficient numbers, particularly those diagnosed after the age of 30 years. Verifying this decreased DR15-DQ6 protection with increasing age of onset is essential prior to future mechanistic studies aiming to identify which specific genetic, immunological and environmental factors associated with being younger, enhance this protection.

We aimed to take advantage of recent cohorts with large numbers of adult type 1 diabetes cases, to characterise genetic protection associated with DR15-DQ6 in later life. Within a type 1 diabetes cohort study (After Diabetes Diagnosis Research Support System-2 [ADDRESS-2]) and UK Biobank population cohort we evaluate the impact of the DR15-DQ6 genotype on type 1 diabetes risk throughout the first five decades of life.

## Methods

In two cohorts (a UK wide type 1 diabetes cohort study, ADDRESS-2, and the UK Biobank), we assessed the proportion of the type 1 diabetes population with DR15-DQ6 relative to control populations, comparing the proportion in those diagnosed in childhood (age ≤ 18 years) and young adulthood (age > 18 years to ≤30 years) to those diagnosed in older adulthood (age > 30 to ≤50 years).

### Participants and diabetes classification

All study participants gave informed consent or assent for underage participants and the study was carried out in accordance with the Declaration of Helsinki as revised in 2008.

#### ADDRESS-2

ADDRESS-2 is a study of incident cases of type 1 diabetes from across the UK within 6 months of diagnosis and 5 years of age or older at recruitment [10]. We studied a subset of 1458 people who self-reported as being of white European ethnicity and had type 1 diabetes defined by ≥1 islet-specific autoantibody, had a clinician diagnosis of type 1 diabetes confirmed within 12 months of onset, and were between the age of 5 and 50 years. Characteristics of people who met the inclusion criteria are shown in Table 1. In those aged 18 years or younger at recruitment, BMI was age adjusted using WHO (2007) reference data [11].

**Table 1:** Characteristics of those meeting study criteria for type 1 diabetes in groups diagnosed above and below 30 years of age in the ADDRESS-2 study

Characteristic	Age ≤ 30 years at diagnosis (*N* = 1097)	Age 31–50 years at diagnosis (*N* = *361*)	*p* value
Age, years	16 (12–23)	38 (33–43)	<0.0001
BMI^a^, kg/m^2^	23.0 (20.8–25.6)	24.8 (22.3–27.8)	<0.0001
T1DGRS	0.275 (0.257–0.295)	0.273 (0.256–0.290)	0.03
Sex, % male	58 (55, 61)	63 (58, 68)	0.1
≥2 Autoantibodies, %	76 (74, 79)	60 (55, 65)	<0.0001

Data are presented as median (IQR) or % (95% CI)

^a^BMI was derived as a *z* score for children using WHO (2007) reference data

#### UK Biobank

We studied 2502 people with diabetes in UK Biobank who were of genetically defined white European ethnicity [12], self-reported a diagnosis of diabetes when aged 50 years or younger, and were insulin treated. Diabetes type is not well defined in UK Biobank, with neither C-peptide nor islet autoantibodies measured. Previous work has shown that within UK Biobank the variable ‘insulin treatment’ is sensitive but not specific for type 1 diabetes [13]. We studied insulin-treated individuals to increase the relative proportion of type 1 diabetes to type 2 diabetes in our cohort and therefore increasing precision of our genetic excess method to identify type 1 diabetes as previously described [13, 14].

#### Control populations

We used HLA allele frequencies from 355,258 people genetically defined as being white European, not reporting diabetes at recruitment. Control individuals with type 2 diabetes were defined based on the presence of self-reported diabetes not currently insulin treated and were genetically defined as being white European (*N* = 14,139).

### HLA genotypes

We assessed the frequency of common type 1 diabetes risk or protection genotypes centrally imputed by UK Biobank to a combination of the Haplotype Reference Consortium (HRC) panel and the UK10K plus 1000 Genomes Project panels [12]. These included risk-reducing DR15-DQ6 (DRB1*15-DQA1*01-DQB1*06) marked by rs3129889 in both datasets as described elsewhere [15]. DR3-DQ2 (DRB1*03:01-DQA1*05:01-DQB1*02:01) and DR4-DQ8 (DRB1*04-DQA1*03-DQB1*03:02) were marked by rs2187668 and rs7454108, respectively. Other possible HLA haplotypes (denoted DRX) were considered to be of neutral risk for type 1 diabetes. SNPs were directly genotyped in ADDRESS-2 as previously described [16], and for UK Biobank were determined from UK Biobank Affymetrix Axiom Array data as previously described [12]. We focused our comparison of diabetes incidence in three groups of people who had one or more alleles indicating the presence of DR15-DQ6, or a single allele indicating the presence of either DR3-DQ2 or DR4-DQ8.

### Non-HLA type 1 diabetes genetic risk score

A non-HLA type 1 diabetes genetic risk score (T1DGRS) was generated from 32 non-HLA loci independently associated with type 1 diabetes risk (receiver operating characteristic AUC for type 1 diabetes vs control 0.72), as previously described [17] (ESM Table 1). A reference non-HLA T1DGRS for type 1 diabetes was derived from cases (*n* = 6483) taken from the Diabetes Genetics Consortium (T1DGC) [15].

### Type 2 diabetes genetic risk score

A type 2 diabetes genetic risk score (T2DGRS) was generated using published variants known to be associated with risk of type 2 diabetes [18, 19]. We generated a 77 SNP T2DGRS in both the Wellcome Trust Case Control Consortium cohort and UK Biobank consisting of variants present in both datasets and with high imputation quality (*R*^2^ > 0.4). The score was generated by summing the effective allele dose of each variant multiplied by the natural log (log_*e*_) of the OR. A reference score for the T2DGRS in type 1 diabetes was derived from cases (*n* = 1847) taken from the Wellcome Trust Case Control Consortium [20].

### Statistical analysis

We included all 357,760 white European individuals from UK Biobank (2502 insulin-treated diabetes cases and 355,258 controls) as a population at risk of developing type 1 diabetes. As HLA genotype is unchanged from birth, we were able to stratify individuals according to HLA type and model diabetes incidence with age as the timescale and diabetes diagnoses as the outcome of interest. Each eligible individual was followed from birth until the earliest of the following: (1) their age at last UK Biobank review; (2) age 50 years; or (3) if diagnosed with diabetes, the age at which they were diagnosed. Follow-up for each individual was split into up to three distinct age of diagnosis categories: childhood (18 years and under); young adulthood (19–30 years inclusive); and older adulthood (31–50 years inclusive). We then calculated the incidence of diabetes for each HLA group within each age category using Poisson regression, modelled with an HLA group by age category interaction term (to specifically test if age category affected HLA-associated risk).

For each age category, we estimated the difference in the estimated diabetes incidence rates for HLA categories DR3-DQ2 or DR4-DQ8 and DR15-DQ6 compared with baseline (DRX). We assumed the incidence of type 2 diabetes in these HLA categories was constant and that differences in incident rates of diabetes relative to DRX reflected differences in type 1 diabetes risk.

After the age of 50 years, over 95% of people in UK Biobank who develop diabetes have type 2 diabetes [13]. The combination of a relatively small number of type 1 diabetes cases and the very high proportion who have type 2 diabetes, means the subtraction analysis stratified by HLA category is imprecise [14]. We therefore censored data at age 50 years. We restricted our primary analysis in ADDRESS-2 to people of the same age to allow direct comparison, and performed a sensitivity analysis on those diagnosed over the age of 50 years (*n* = 80).

We further tested the assumption that most DR15-DQ6-associated diabetes, even if insulin treated, is type 2 diabetes as a result of dominant protection from type 1 diabetes. We assessed non-HLA T1DGRS and a T2DGRS in people with insulin-treated diabetes who had one or more copies of DR15-DQ6. We hypothesised that if DR15-DQ6 is protective against type 1 diabetes, people with insulin-treated diabetes and one or more copy of DR15-DQ6 would have both non-HLA type 1 diabetes and type 2 diabetes genetic risk identical to a type 2 diabetes cohort.

Allele frequencies and ORs in both UK Biobank and ADDRESS-2 were adjusted against population frequencies derived from white UK Biobank participants without diabetes. These were consistent with previously reported frequencies from white European populations [3, 4]. In ADDRESS-2, ORs were calculated using two-by-two contingency tables with χ^2^ analysis used for comparisons of difference for categorical characteristics and for continuous data we performed unpaired *t* tests for significance. All analyses were performed using Stata 16 (StataCorp LP, College Station, TX, USA).

## Results

### DR15-DQ6 is protective for type 1 diabetes, defined by clinical diagnosis and autoantibody positivity, throughout the first five decades of life in ADDRESS-2

**Fig. 1:**
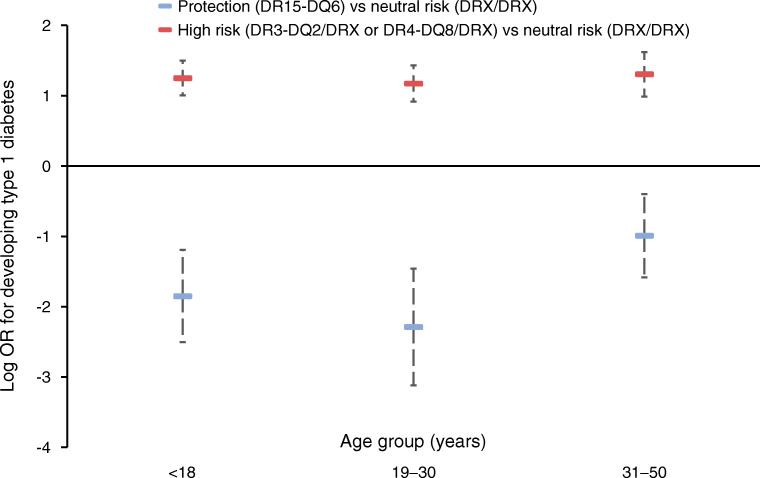
Comparison of risk HLA genotype vs protective HLA genotype log_*e*_ ORs for developing type 1 diabetes defined by clinical diagnosis and autoantibody positivity within the first five decades of life within the ADDRESS-2 study. The highest risk compound heterozygote and homozygote risk HLA genotypes are shown in Table 2 for comparison. Bars represent 95% CIs

Relative to a neutral HLA genotype there was a maintained reduction in risk for type 1 diabetes afforded by DR15-DQ6 in all age groups (all OR < 1, *p* < 0.001). An attenuation of this protection was seen with increasing age of diagnosis of type 1 diabetes (age 5–18 years OR 0.16 [95% CI 0.08, 0.31] and age 19–30 years OR 0.10 [95% CI 0.04, 0.23] vs age 31–50 years OR 0.37 [95% CI 0.21, 0.68], *p* = 0.003). The risk of type 1 diabetes afforded by DR3-DQ2 and DR4-DQ8 remained constant throughout the age range evaluated (all *p* > 0.5) (Fig. 1 and Table 2). A sensitivity analysis to evaluated the risk of those diagnosed over 50 years of age (*n* = 80) developing type 1 diabetes, showed that DR15-DQ6 remained protective (OR 0.29) at a median age of 56 years (IQR 53–59) (*p* = 0.01). In all age groups, participants homozygous for DR3-DQ2 or DR4-DQ8 or compound heterozygous for both risk alleles showed considerable increased risk for type 1 diabetes (ESM Table 2).

**Table 2:** Comparisons of risk vs protective HLA genotype ORs for developing type 1 diabetes defined by clinical diagnosis and autoantibody positivity within the first five decades of life within the ADDRESS-2 study

HLA genotype	Age ≤ 18 years	Age 19–30 years	Age 31–50 years
DR15-DQ6	0.16 (0.08, 0.31)	0.10 (0.04, 0.23)	0.37 (0.21, 0.68)
DR3-DQ2/DRX	2.33 (1.74, 3.12)	2.27 (1.67, 3.08)	2.98 (2.08, 4.27)
DR4-DQ8/DRX	5.48 (4.20, 7.14)	4.89 (3.69, 6.48)	4.99 (3.51, 7.09)
DR3-DQ2/DR3-DQ2	12.96 (9.48, 17.72)	9.16 (6.40, 13.12)	15.27 (10.35, 22.53)
DR4-DQ8/DR4-DQ8	25.36 (17.98, 35.78)	13.89 (8.97, 21.52)	20.98 (13.07, 33.69)
DR3-DQ2/DR4-DQ8	27.90 (21.53, 36.15)	15.17 (11.20, 20.55)	18.12 (12.58, 26.09)

Data are OR (95% CI) calculated relative to DRX/DRX

DRX denotes neutral HLA

### DR15-DQ6 is dominantly protective for type 1 diabetes throughout the first five decades of life relative to a neutral HLA genotype within UK Biobank

**Fig. 2:**
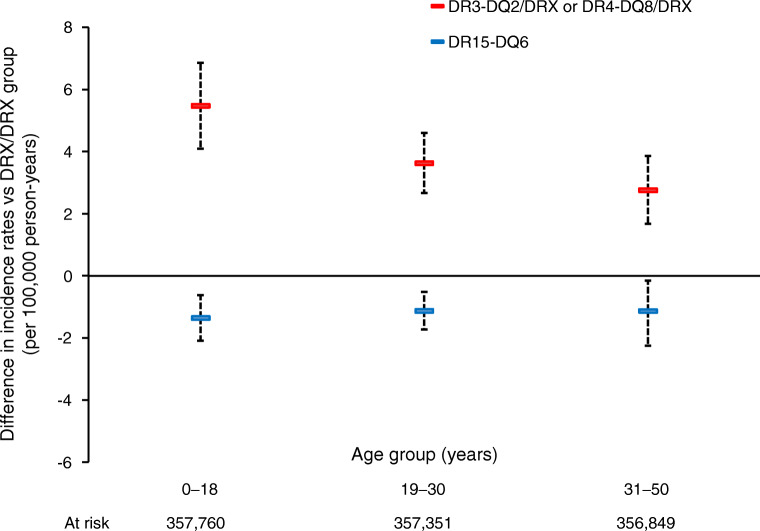
The difference in incidence of diabetes per 100,000 person-years in different HLA genotypes relative to a neutral HLA genotype within the UK Biobank. Bars represent 95% CIs

DR15-DQ6 was associated with decreased risk of diabetes relative to a neutral HLA genotype throughout the first five decades of life in UK Biobank (Fig. 2). This protection was dominant as it was unaffected by the corresponding allele at the same locus being neutral (DRX), increasing risk for type 1 diabetes (DR3-DQ2 or DR4-DQ8), or being a second copy of DR15-DQ6 (all *p* > 0.1) (ESM Figs 1, 2). This result was similar across all three age groups. As expected, all HLA risk alleles were associated with increased diabetes risk compared with a neutral HLA genotype in UK Biobank (ESM Table 3).

### Within UK Biobank people with DR15-DQ6 and diabetes have non-HLA T1DGRS and T2DGRS consistent with having type 2 diabetes

**Fig. 3:**
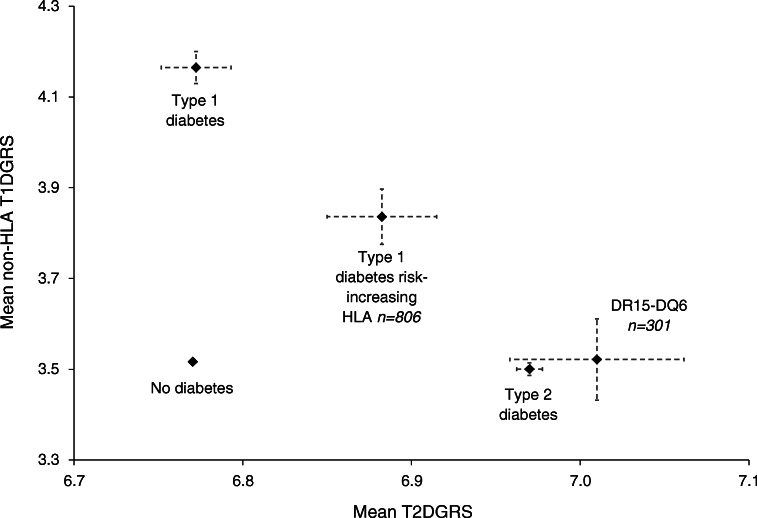
Mean non-HLA T1DGRS plotted against mean T2DGRS within individuals with insulin-treated diabetes in UK Biobank diagnosed at aged 31–50 years stratified by HLA genotype group. Individuals with one or two copies of DR15-DQ and those with type 1 diabetes risk-increasing genotypes (presence of DR3 and/or DR4-DQ8) are shown. Cohorts with type 1 diabetes, type 2 diabetes and controls without diabetes are plotted for reference. Bars represent 95% CIs

We evaluated a non-HLA T1DGRS and a T2DGRS, stratified by HLA group, in our cohort of individuals with insulin-treated diabetes aged 31–50 years within UK Biobank (Fig. 3). The 301 individuals with insulin-treated diabetes and one or two DR15-DQ6 haplotypes had a similar mean non-HLA T1DGRS and a similar T2DGRS to individuals with type 2 diabetes (each *p* > 0.1). This suggests almost all people with insulin-treated diabetes and one or two DR15-DQ6 haplotypes have type 2 diabetes. People with HLA genotypes consistent with raised type 1 diabetes risk (containing DR3-DQ2 and/or DR4-DQ8) showed intermediate non-HLA type 1 diabetes and type 2 diabetes genetic risk relative to type 1 diabetes and type 2 diabetes reference cohorts. This indicates that our selection criteria, of diagnosis between the ages of 31 and 50 years and insulin treatment, includes a mixture of both type 1 diabetes and type 2 diabetes cases (all associations *p* < 0.001). In those with insulin-treated diabetes who were diagnosed at age ≤30 years, the non-HLA T1DGRS and T2DGRS of HLA genotypes associated with increased type 1 diabetes risk were comparable with a reference type 1 diabetes cohort (ESM Fig. 3) (*p* > 0.05), consistent with almost all of this cohort having type 1 diabetes.

## Discussion

We have demonstrated that the protection afforded by DR15-DQ6 against type 1 diabetes persists across the first five decades of life, in a dominant manner. The reduction in diabetes risk with DR15-DQ6 was observed in two separate cohorts and irrespective of whether type 1 diabetes was defined with a combination of clinical classification and autoantibody positivity or it was stratified genetically.

The dominant nature of DR15 et alDQ6 protection we describe is consistent with numerous previous studies in children [5]. Despite DR15-DQ6 being extremely common (~15–20%) in European populations, it is largely absent in cohorts of children with type 1 diabetes, being found in just 1–2% of these children [5–7]. The overall protective effect of DR15 in adults seen in our studies is consistent with these childhood findings, with just 2.7% of those diagnosed over 18 years of age with positive islet autoantibodies in the ADDRESS-2 cohort having DR15-DQ6. The high frequency of DR15-DQ6 in European populations combined with the very strong protection described above are contributing factors to the efficacy of genetic risk scores in these populations [17, 21].

Our finding that the protection against type 1 diabetes conferred by DR15-DQ6 is preserved with increasing age helps resolve an outstanding question around genetic protection from type 1 diabetes and whether it persists into adulthood. Graham et al [8] studied a cohort of 830 participants with diabetes from Sweden and observed a loss of DR15-DQ6 protection by 35 years of age relative to a neutral DRX homozygous genotype (OR 1.1 by 35 years of age). They described a continuous reduction in protection (OR 0.09 in those diagnosed at age 7–13 years rising to 0.57 in those diagnosed at 28 and 34 years of age). In contrast, Howson et al [9] studied type 1 diabetes-associated HLA and non-HLA variants in 1384 adults (median age 31 years) with type 1 diabetes. Their study showed very low allele frequency for DR15-DQ6 (tagged by rs9271366), suggesting ongoing reduced type 1 diabetes risk associated with DR15-DQ6.

In our study we were able to analyse the effect of DR15-DQ6 in over 750 individuals who developed type 1 diabetes at age >30 years [13]. We studied people up to the age of 75 years in ADDRESS-2 and observed protection from type 1 diabetes throughout all age ranges, with the OR for DR15-DQ6 still strongly protective in the 80 people diagnosed at age 50–75 years. Additionally, we showed this protection was equivalent whether DR15-DQ6 was heterozygous or homozygous and was maintained in the presence of a copy of DR3-DQ2 or DR4-DQ8, a question Howson et al’s study did not directly address.

The main difference between all the studies was how type 1 diabetes was defined. In the Swedish study, diabetes was classified clinically and without use of islet autoantibodies; in contrast, Howson et al’s and our study both used robust definitions of type 1 diabetes. The observed lesser protection with increasing age reported by Graham et al could reflect misdiagnosis, with older clinically diagnosed type 1 diabetes cohorts more likely to contain individuals with type 2 diabetes [22, 23]. Taken together, these findings from three separate studies (Howson et al, ADDRES-2 and UK Biobank) highlight the possibility that strong genetic protection from type 1 diabetes exists across all ages of diagnosis and that previous observations of reducing protection with age are either much more modest or non-existent. This notion is further supported by a study by Caillat-Zucman et al [24] showing mildly attenuated but maintained protection against type 1 diabetes in a sample of 290 adults (mean age at diagnosis 27 years) with *HLA-DR15-DQ6*. Callait Zucman et al did not observe the dominant protection that we describe and this may relate to our larger sample size. Caillat-Zucman et al also first described the reduction in *HLA-DR3-DQ2/DR4-DQ8* with increasing age of type 1 diabetes onset, a finding replicated in our study between children and young adults. Between adult groups we did not see a large reduction in risk of type 1 diabetes associated with *HLA-DR3-DQ2/DR4-DQ8* as previously reported [24]; however, there were wide CIs around these estimates.

Our study has some limitations. We only studied people of white European ethnicity and findings should not be extrapolated to non-white ethnicities without studying these populations, particularly as the allele frequency of DR15-DQ6 is lower in other ethnicities [21]. A genetic variant, marked by allele 15 at the *D6S265* locus, has previously been shown to alter genetic risk related to DR15-DQ6 [25]. We were not able to characterise this microsatellite variant as it was not genotyped in our study but it is possible that further characterisation of extended DR15-DQ6 haplotypes may improve risk stratification related to this haplotype. Our results in UK Biobank would be strengthened by islet autoantibody or C-peptide data from individuals with diabetes but, like many large population biobanks, these were not measured. Reassuringly, our results between UK Biobank and ADDRESS-2, a study using a clinical diagnosis of type 1 diabetes coupled to islet autoantibody positivity, were consistent.

We did not perform high-resolution HLA typing of our cohorts, so our results are based on an assumption that the SNPs we used accurately tag DR3-DQ2, DR4-DQ8 and DR15-DQ6. Although established reference cohorts with both SNP array genotyping and HLA typing support the efficacy of the SNP tags used, findings may not extend to non-white populations where HLA linkage disequilibrium structures vary and certain DRB1*04 subtypes, including those with a protective effect, may be more common.

## Conclusion

Our study shows that the protection against type 1 diabetes afforded by DR15-DQ6 is strong and persists in a dominant fashion across the first five decades of life in a cohort of white Europeans. Understanding the mechanisms underpinning this protection offers exciting potential therapeutic avenues for type 1 diabetes.

## Supplementary Information

ESM (PDF 623 kb)

## Data Availability

ADDRESS-2 data access is available via a management committee [10]. UK Biobank data are available through a procedure described at http://www.ukbiobank.ac.uk/using-the-resource/.

## Abbreviations

T1DGRS: Type 1 diabetes genetic risk score
T2DGRS: Type 2 diabetes genetic risk score
