# Supplementary material for: DR15-DQ6 remains dominantly protective against type 1 diabetes throughout the first five decades of life
Source: Diabetologia. 2021 Jul 16;64(10):2258–65. doi: 10.1007/s00125-021-05513-4 (PMC8423681; doi:10.1007/s00125-021-05513-4)
Supplement: Supplementary file 1 — (PDF 623 kb) [file 125_2021_5513_MOESM1_ESM.pdf]

Electronic supplementary material (ESM)

Gene	SNP	Odds Ratio	Beta	Allele	Source
INS	rs3842753	2.29	0.83	G	Internal
PTPN22	rs2476601	1.89	0.64	A	Onengut
CTSH	rs2289702	1.32	0.28	C	Internal
ATXN2	rs653178	1.30	0.26	C	Onengut
COBL	rs4948088	1.30	0.26	C	Winkler
IL27	rs9924471	1.24	0.22	A	Poicot
ERBB3	rs4759229	1.24	0.22	A	Internal
PTPN2	rs1893217	1.21	0.19	G	Onengut
BACH2	rs72928038	1.20	0.18	A	Onengut
RNLS	rs60888743	1.20	0.18	A	Internal
ITGB7	rs11170466	1.19	0.17	T	Poicot
UBASH3A	rs9981624	1.19	0.17	C	Interal
CENPW	rs9388489	1.17	0.16	A	Onengut
HORMAD2	rs5763779	1.16	0.15	A	Internal
PRKD2	rs425105	1.16	0.15	T	Onengut
RASGRP1	rs72727394	1.15	0.14	T	Onengut
ADAD1	rs17388568	1.13	0.12	A	Poicot
CD226	rs1615504	1.13	0.12	T	Onengut
COBL	rs6476839	1.12	0.11	T	Onengut
IRF7	rs9585056	1.12	0.11	C	Onengut
C1QTNF6	rs229541	1.11	0.10	A	Poicot
SIRPG	rs2281808	1.11	0.10	C	Steck
TAGAP	rs1738074	0.92	-0.08	T	Poicot
MEG3	rs56994090	0.88	-0.13	C	Onengut
CLEC1	rs10492166	0.87	-0.14	A	Poicot
PTPN22	rs3024505	0.86	-0.15	A	Onengut
IFIH1	rs2111485	0.85	-0.16	A	Onengut
CTLA4	rs3087243	0.84	-0.17	A	Onengut
CLEC16A	rs12708716	0.83	-0.19	G	Poicot
TYK2	rs144309607	0.67	-0.40	T	Onengut
IL2RA	rs61839660	0.62	-0.48	T	Onengut
IL2RA	rs41295121	0.49	-0.71	T	Onengut

ESM table 1. 32 SNPs to generate non-HLA type 1 diabetes grs.

		Age groups (years)		
**HLA *n* (% of age group)**	**Controls**	**≤ 18**	**19-30**	**31-50**
**DRX/DRX**	132,399	86 (13)	80 (17)	51 (14)
**DR15-DQ6**	97,071	10 (2)	6 (1)	14 (4)
**DR3-DQ2/DRX or DR4-DQ8/DRX**	104319	243 (38)	209 (46)	152 (42)
**DR3-DQ2/DR3-DQ2, DR4-DQ8/DR4-DQ8 or DR3-DQ2/DR4-DQ8**	21486	300 (47)	163 (36)	144 (40)
**Odds Ratio (95% CI), calculated relative to DRX/DRX**				
**DR15-DQ6**	1.0	0.2 (0.1, 0.3)	0.1 (0.0, 0.2)	0.4 (0.2, 0.7)
**DR3-DQ2/DRX or DR4-DQ8/DRX**	1.0	3.6 (2.8, 4.6)	3.3 (2.6, 4.3)	3.8 (2.8, 5.2)
**DR3-DQ2/DR3-DQ2, DR4-DQ8/DR4-DQ8 or DR3-DQ2/DR4-DQ8**	1.0	21.5 (16.9, 27.3)	12.6 (9.6, 16.4)	17.4 (12.6, 24.0)

ESM Table 2: Odds ratio's (OR) for developing type 1 diabetes defined by autoantibody positivity by HLA genotype relative to UK Biobank controls without diabetes.

			[95% Conf.Interval]	
**Age group (years)**	**HLA group**	**Difference in incidence rates relative to DRX/DRX (per 100,00 person-years)**	**Lower**	**Upper**
**0-18**	DR3-DQ2/DRX or DR4-DQ8/DRX	5.48	4.1	6.86
**19-30**	DR3-DQ2/DRX or DR4-DQ8/DRX	3.63	2.67	4.6
**31-50**	DR3-DQ2/DRX or DR4-DQ8/DRX	2.76	1.67	3.86
**0-18**	DR15-DQ6	-1.35	-2.09	-0.619
**19-30**	DR15-DQ6	-1.12	-1.73	-0.52
**31-50**	DR15-DQ6	-1.13	-2.252	-0.19
**0-18**	DR3-DQ2/DR3-DQ2, DR4-DQ8/DR4-DQ8 or DR3-DQ2/DR4-DQ8	46.62	39.74	53.5
**19-30**	DR3-DQ2/DR3-DQ2, DR4-DQ8/DR4-DQ8 or DR3-DQ2/DR4-DQ8	26.9	22.77	31.04
**31-50**	DR3-DQ2/DR3-DQ2, DR4-DQ8/DR4-DQ8 or DR3-DQ2/DR4-DQ8	18.99	15.84	22.15

ESM Table 3. Difference in incidence rates relative to a neutral (XX) genotype in UK Biobank.

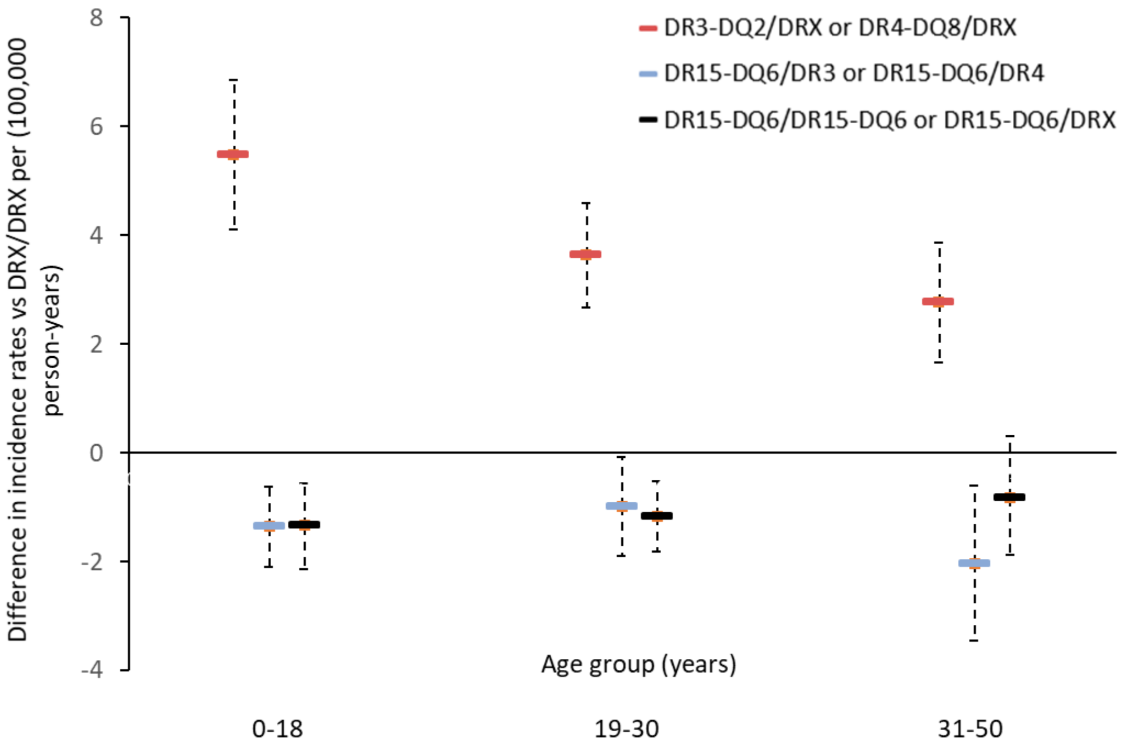

ESM Figure 1: The impact of the addition of a risk (DR3 or DR4) versus neutral (DRX) to the protective DR15-DQ6 allele at the corresponding locus.

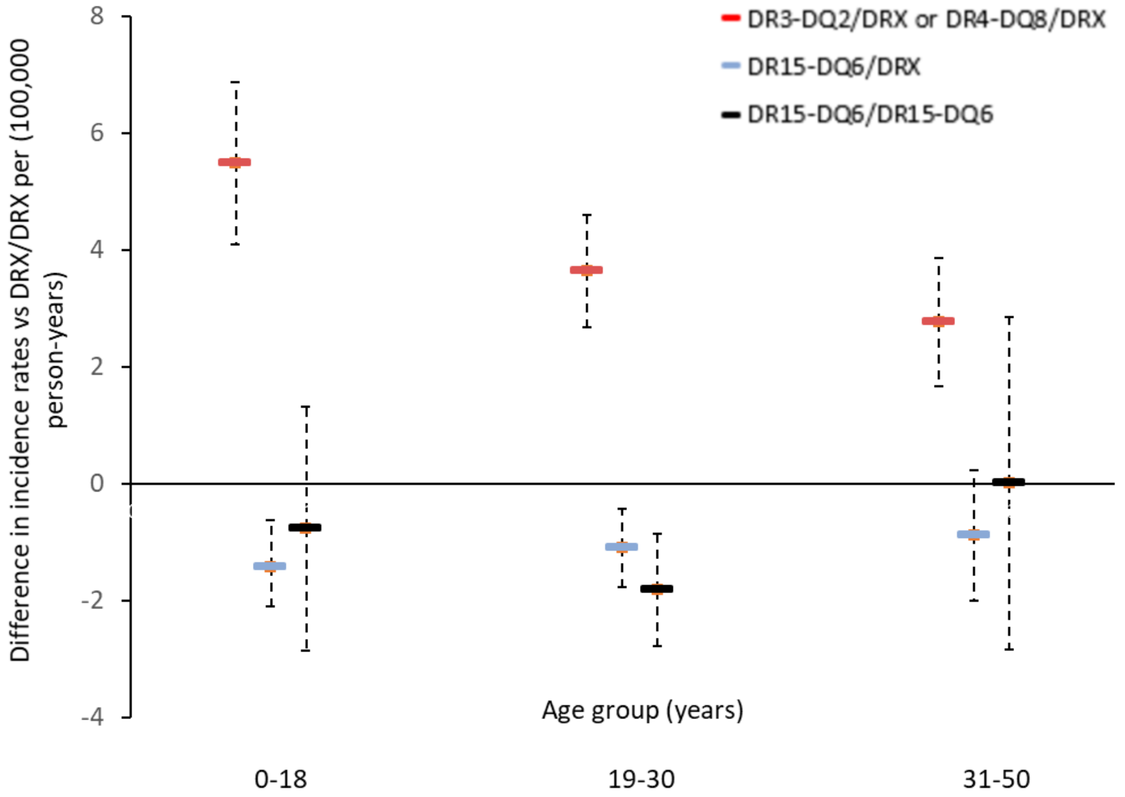

ESM figure 2: *The impact of the substitution of a further protective DR15-DQ6 allele for a neutral (DRX) when a DR15-DQ6 allele is already present at the corresponding locus.*

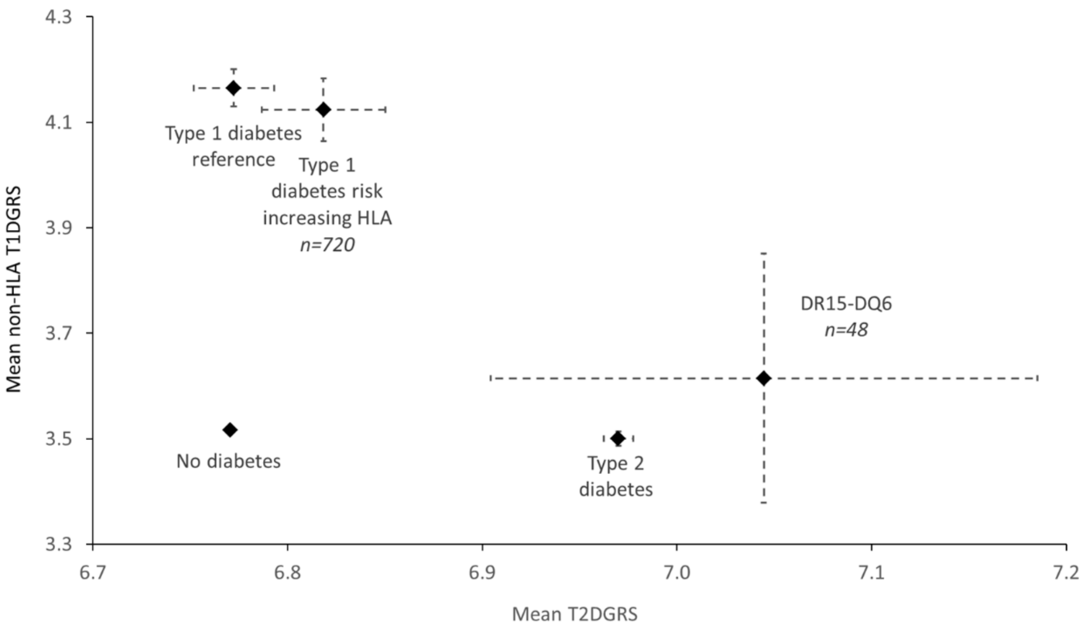

ESM Figure 3. Means and 95% confidence intervals of non-HLA type 1 diabetes genetic risk score (non-HLA TIDGRS) plotted against mean type 2 diabetes genetic risk score (T2DGRS) within insulin treated diabetes in UK Biobank diagnosed aged ≤30 stratified by HLA genotypes group. Individuals with one or two copies of DR15-DQ6, type 1 diabetes risk increasing genotypes (presence of DR3 and/or DR4-DQ8). Cohorts with type 1 diabetes type 2 diabetes and controls without diabetes are plotted for reference. Bars represent 95% confidence intervals.
